# Supplementary material for: A randomized multiplex CRISPRi-Seq approach for the identification of critical combinations of genes
Source: bioRxiv. 2023 Aug 26:2023.02.03.527066. Originally published 2023 Feb 3. Preprint. [Version 2] doi: 10.1101/2023.02.03.527066 (PMC10028747; doi:10.1101/2023.02.03.527066)
Supplement: Supplementary file 1 [file NIHPP2023.02.03.527066v2-supplement-2.pdf]

bioRxiv preprint doi: https://doi.org/10.1101/2023.02.03.527066; this version posted August 26, 2023. The copyright holder for this preprint (which was not certified by peer review) is the author/funder. This article is a US Government work. It is not subject to copyright under 17 USC 105 and is also made available for use under a CC0 license.

## Supplemental Information

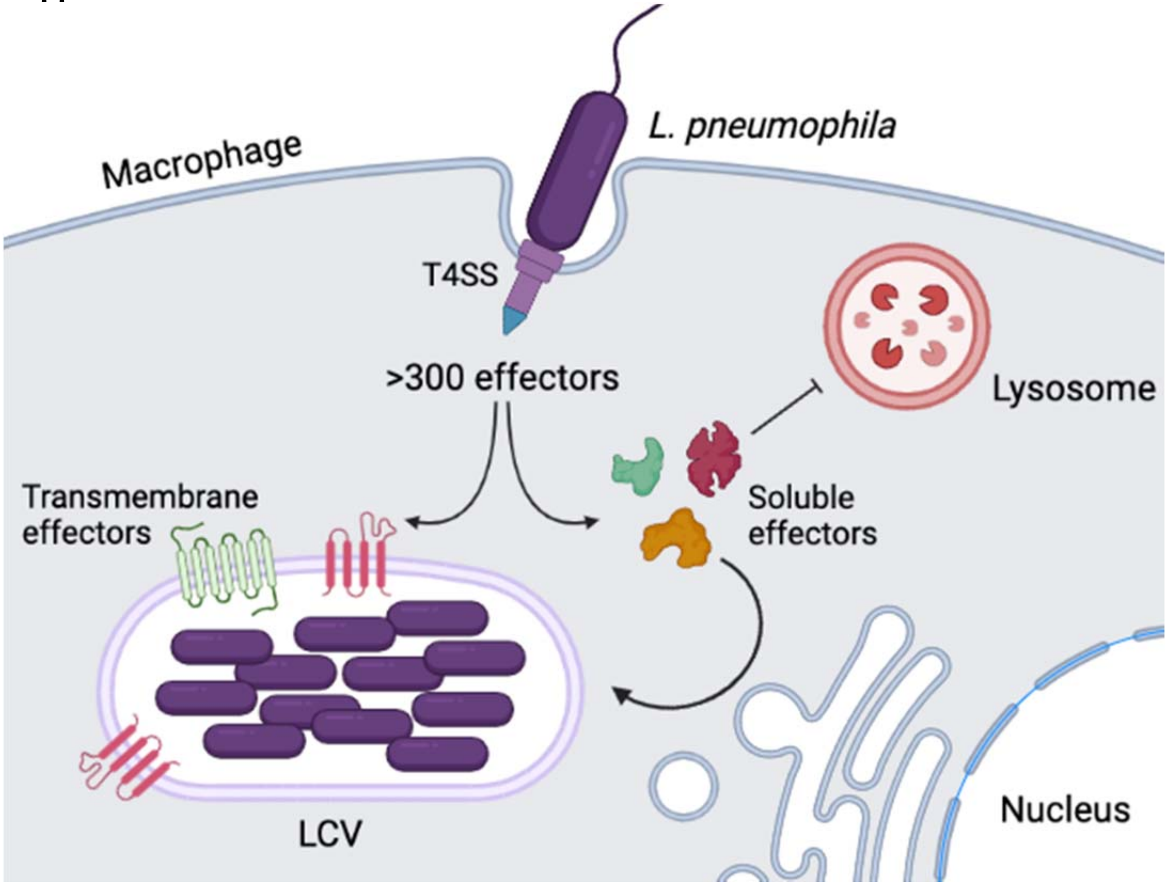


**Figure S1: Model of the *Legionella*-containing vacuole bearing transmembrane effectors.** *L. pneumophila* translocates >300 effector proteins *via* a T4SS into a host cell to re-wire cellular pathways to evade the lysosome and establish a replication vacuole known as the *Legionella*-containing vacuole (LCV). It is hypothesized that transmembrane effectors are incorporated into the LCV for nutrient acquisition, detoxification, and membrane fusion. Diagram made using BioRender (https://biorender.com).

bioRxiv preprint doi: https://doi.org/10.1101/2023.02.03.527066; this version posted August 26, 2023. The copyright holder for this preprint (which was not certified by peer review) is the author/funder. This article is a US Government work. It is not subject to copyright under 17 USC 105 and is also made available for use under a CC0 license.

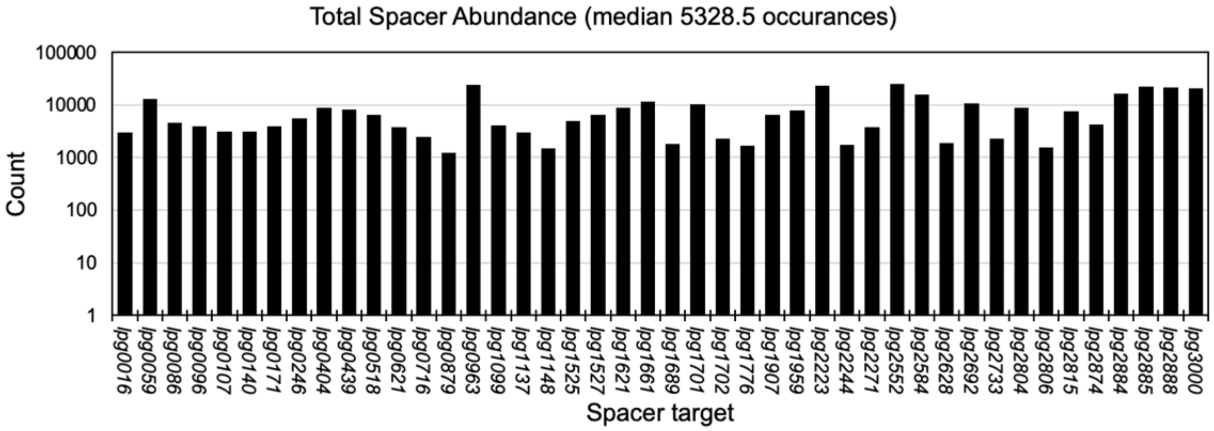


**Figure S2: Spacer abundance post CRISPRi induction.**

The occurrence of each spacer within the plasmid libraries was quantified 24 hours post induction of expression. The fact that each spacer was well represented within the library indicated that the crRNA products of these spacers were not toxic to axenic *L. pneumophila* growth.

**Note S1: *In vitro* CRISPR array assembly**

The *in vitro* assembly of crRNA-encoding arrays is a key accomplishment of our approach. While it seems simple on paper, the actual assay proved to be technically demanding and required various optimization steps. For experimenters interested in applying our technology to their own research question, the following is a list of strategies we also tried:

**Size selection of CRISPR arrays:** To enrich for longer CRISPR arrays, we explored use of size-selection SPRI beads (Beckman), Pippin Prep instrumentation (Sage Science), and immediate purification from the R-S-R assembly mix by DNA gel electrophoresis. The goal was to maintain the most ligated material and give preference to longer arrays that are outcompeted by smaller arrays during vector incorporation.  While these approaches were able to size-select for longer arrays, the highest yield of size-selected arrays occurred when the ligated arrays were first

bioRxiv preprint doi: https://doi.org/10.1101/2023.02.03.527066; this version posted August 26, 2023. The copyright holder for this preprint (which was not certified by peer review) is the author/funder. This article is a US Government work. It is not subject to copyright under 17 USC 105 and is also made available for use under a CC0 license.

introduced into an interim plasmid, then excised *via* restriction enzyme digest, size-ordered by gel electrophoresis, extracted from the gel, and then ligated back into the donor plasmid.

**Addition of promoter and terminator:** Attempts to add dead ends bearing the promoter and terminator sequences to the original R-S-R assembly mix led to muddled array assembly which we believed to be the result of the promoter and terminator fragments being much longer (~200 bps) than the R-S-R building blocks (60 bps) Therefore, addition of the promoter and terminator to the final plasmid was accomplished by Invitrogen Multisite Gateway Pro cloning as described.

**Barcoding of arrays:** Barcodes are often used to distinguish between different constructs in library-based experiments. The pooled nature of our *de novo* array self-assembly protocol thwarts unique barcode addition without interim isolation of each array followed by long-read sequencing to assign each array to a unique bar code. Since long-read sequencing by PacBio sequences each array in its entirety, our MuRCiS pipeline negates the need for barcodes altogether.

**Sub-optimal annealing sites:** During the development of our MuRCiS workflow, we also made an array library in which we intentionally designed spacers to encode crRNAs that would target sequences adjacent PAMs further downstream of the transcriptional start site. We hoped they would serve as good off-targeting controls. However, upon performing the infection experiment, we found almost none of the hits that emerged while using the most optimal spacers (Figures 5 and 6) and so we would recommend only using the best crRNA target.

bioRxiv preprint doi: https://doi.org/10.1101/2023.02.03.527066; this version posted August 26, 2023. The copyright holder for this preprint (which was not certified by peer review) is the author/funder. This article is a US Government work. It is not subject to copyright under 17 USC 105 and is also made available for use under a CC0 license.

**Supplemental Tables**

Table S1: 10-plex Construct Descriptions and Spacer Sequences

Table S2: TME Genes and Corresponding Spacer Sequences

Table S3: PacBio Long-read Sequencing Metrics

Table S4: Read Counts and Calculations for all Constructs from all Experiments

Table S5: Subset data for all hits identified in U937 macrophage infection experiments

Table S6: Subset data for all critical gene combinations identified in U937 macrophage infection experiments

Table S7: Subset data for all hits identified in *A. castellanii* infection experiments

Table S8: Subset data for all critical gene combinations identified in *A. castellanii* infection experiments

Table S9: Hits found in single round CRISPRi experiments

Table S10: Strains and Plasmids Used in this Study
